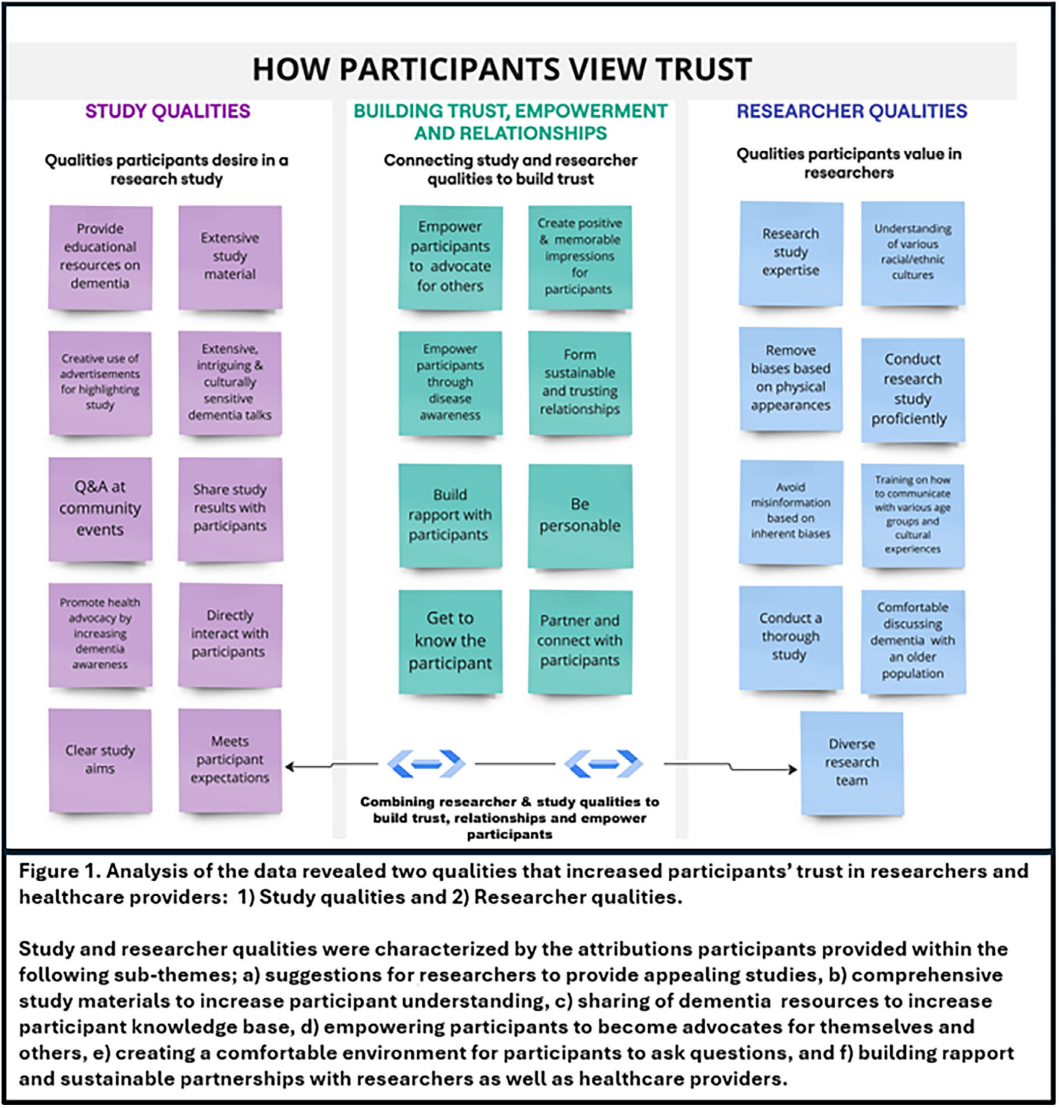# Black American's views on trust

**DOI:** 10.1002/alz70858_100415

**Published:** 2025-12-25

**Authors:** Shenikqua Bouges, Esra Alagoz, Diana Gutierrez‐Meza, Gilda E. Ennis, Taryn T. James, Diane Carol Gooding, Carol A. Van Hulle, Nickolas H Lambrou, Susan Flowers Benton, Debra Noell, Alayna Oby, Fauzia Hollnagel, Barbara L. Fischer, Fabu P. Carter, Carey E. Gleason

**Affiliations:** ^1^ Wisconsin Alzheimer's Disease Research Center, University of Wisconsin School of Medicine and Public Health, Madison, WI, USA; ^2^ Geriatric Research, Education and Clinical Center (GRECC), William S. Middleton Memorial Veterans Hospital, Madison, WI, USA; ^3^ Division of Geriatrics and Gerontology, University of Wisconsin‐Madison, School of Medicine and Public Health, Madison, WI, USA; ^4^ University of Wisconsin ‐ Madison, Madison, WI, USA; ^5^ Department of Psychology, University of Wisconsin‐Madison, College of Letters & Science, Madison, WI, USA; ^6^ Southern University and A&M College, Baton Rouge, LA, USA; ^7^ University of Wisconsin ‐ Madison, MADISON, WI, USA; ^8^ William S. Middleton Memorial Hospital, Madison, WI, USA; ^9^ Department of Biostatistics and Medical Informatics, University of Wisconsin‐Madison SMPH, Madison, WI, USA; ^10^ Department of Neurology, University of Wisconsin‐Madison, School of Medicine and Public Health, Madison, WI, USA; ^11^ Division of Geriatrics and Gerontology, Department of Medicine, University of Wisconsin School of Medicine and Public Health, Madison, WI, USA; ^12^ University of Wisconsin School of Medicine and Public Health, Madison, WI, USA

## Abstract

**Background:**

In the United States, 18.6% of Black Americans over age 65 are impacted by Alzheimer's disease and ∼ 5% participate in clinical trials. Research has shown that limited access to education, information and mistrust in the healthcare system contribute to less representation of Black participants in clinical trials. Mistrust in the healthcare system is a well‐known barrier to clinical trial participation. To gauge Black Americans’ views on trust, our study used the community based participatory research framework to increase dementia awareness, provide services to Black participants and seek the community's views on trust.

**Method:**

We conducted four focus group discussions with Black participants 45 years and older without self‐reported cognitive impairment. Discussions were facilitated by a race concordant moderator and a moderator not identifying as Black via a virtual platform. The transcripts were analyzed by one geriatric physician and two qualitative researchers using inductive thematic analysis. Central themes were identified using narrative summaries and mind maps. Our results show a conceptual framework of how participants view trust.

**Result:**

Participants self‐identified as Black, had a mean age of 63, were predominantly female and completed some college. Our study showed that Black participants developed trust with researchers when they portrayed shared historical experiences, cultural awareness, and had previously established relationships within the community. Participants also valued diverse research teams and researchers with disease expertise. Participants desired to be heard, educated on illnesses affecting their community and informed of comprehensive study results. Data analysis revealed that trust and established relationships with the research team increased participants’ willingness to participate in future studies. See Figure 1

**Conclusion:**

Black participants are willing to participate in research studies, but we as researchers must first create a welcoming aura through partnerships. As we build upon more inclusive research study designs, it will be important for future studies to incorporate relationship building qualities into a standardized inclusive recruitment and retention protocol.